# *In vitro* Analysis of O-Antigen-Specific Bacteriophage P22 Inactivation by *Salmonella* Outer Membrane Vesicles

**DOI:** 10.3389/fmicb.2020.510638

**Published:** 2020-09-24

**Authors:** Mareike S. Stephan, Nina K. Broeker, Athanasios Saragliadis, Norbert Roos, Dirk Linke, Stefanie Barbirz

**Affiliations:** ^1^ Physical Biochemistry, Department for Biochemistry and Biology, University of Potsdam, Potsdam, Germany; ^2^ Department of Biosciences, University of Oslo, Oslo, Norway

**Keywords:** bacteriophage, bacterial outer membrane vesicles, O-antigen, bacterial membrane fractionation, *Salmonella*, lipopolysaccharide

## Abstract

Bacteriophages use a large number of different bacterial cell envelope structures as receptors for surface attachment. As a consequence, bacterial surfaces represent a major control point for the defense against phage attack. One strategy for phage population control is the production of outer membrane vesicles (OMVs). In Gram-negative host bacteria, O-antigen-specific bacteriophages address lipopolysaccharide (LPS) to initiate infection, thus relying on an essential outer membrane glycan building block as receptor that is constantly present also in OMVs. In this work, we have analyzed interactions of *Salmonella* (*S*.) bacteriophage P22 with OMVs. For this, we isolated OMVs that were formed in large amounts during mechanical cell lysis of the P22 S. Typhimurium host. *In vitro*, these OMVs could efficiently reduce the number of infective phage particles. Fluorescence spectroscopy showed that upon interaction with OMVs, bacteriophage P22 released its DNA into the vesicle lumen. However, only about one third of the phage P22 particles actively ejected their genome. For the larger part, no genome release was observed, albeit the majority of phages in the system had lost infectivity towards their host. With OMVs, P22 ejected its DNA more rapidly and could release more DNA against elevated osmotic pressures compared to DNA release triggered with protein-free LPS aggregates. This emphasizes that OMV composition is a key feature for the regulation of infective bacteriophage particles in the system.

## Introduction

Bacteriophages are ubiquitous in the microbial world and major players in many bacterial ecosystems. Here, they perform a variety of life styles, with prophages or mature phage particles as prominent examples of prevalent phage states (Feiner et al., 2015). Key event in a phage’s life cycle is the interaction with host surface receptors to initiate infection of the bacterial host. If this step is successful, the resulting genome transfer to the bacterial cytosol will open the route for a plethora of phage genome functions inside the host cell (Salmond and Fineran, 2015; Erez et al., 2017; Rostol and Marraffini, 2019). Diverse bacterial cell surface structures serve as bacteriophage receptors (Silva et al., 2016). Besides protein-based phage receptors, glycan structures, the major building blocks of the bacterial envelope, represent a very important and structurally diverse phage receptor class (Pires et al., 2016; Latka et al., 2017). Phages do not only exploit glycans to start cell entry (Wang et al., 2019) but also may alter these structures to prevent superinfection (Kintz et al., 2015). In turn, bacteria constantly modify their surface composition to escape from phage attack (Rostol and Marraffini, 2019).

O-antigen-specific phages are specialists that use lipopolysaccharide (LPS) as an essential receptor for infection initiation in Gram-negative hosts (Broeker and Barbirz, 2017). These tailed phages link recognition and enzymatic processing of the O-polysaccharide to a so far unknown membrane interaction step that leads to conformational rearrangements in the tail and subsequent particle opening *in vitro* (Andres et al., 2010, 2012; Broeker et al., 2018, 2019). *In vivo*, full genome transfer additionally requires other factors like outer membrane (OM) proteins or a membrane potential (Leavitt et al., 2013; Parent et al., 2014). *In vitro*, purified and protein-free LPS alone is sufficient to inactivate O-antigen-specific phages, because binding to membrane-mimicking LPS aggregates can trigger DNA release from the capsid (Broeker et al., 2019). *In vitro* studies furthermore revealed that the kinetics of this process were dependent on the architecture of the tail, but not on the chemical composition of the O-antigen receptor. Moreover, to control phage populations and to achieve superinfection exclusion, O-antigen-specific phages may modify the O-antigen structure of their host cell (Susskind et al., 1974; Cenens et al., 2015).

In response to bacteriophage attack, bacteria control the number of phage particles in their environment in multiple ways, and, in turn, phages respond by own strategies to overcome bacterial defense (Doron et al., 2018; Vidakovic et al., 2018; Rostol and Marraffini, 2019). Among the large number of defense mechanisms determining the ecosystem in which bacteria and phages coexist, as a matter of fact, the host surface is a critical point for bacteriophage entry control (Leon and Bastias, 2015). For example, O-antigen phase variations may result in mixed bacterial populations and an increased resistance to phage attack (Seed et al., 2012; Schmidt et al., 2016). Moreover, like all living cells, bacteria can also shed outer membrane vesicles (OMVs; Schwechheimer and Kuehn, 2015). In OMVs, bacteria may control the content of bacteriophage surface receptors and thus exploit this extracellular material as buffer against phage attack (Manning and Kuehn, 2011; Biller et al., 2014; Reyes-Robles et al., 2018).

The ubiquitous budding of spherical OM structures is highly conserved in both pathogenic and non-pathogenic Gram-negative bacteria (Bai et al., 2014; Celluzzi and Masotti, 2016; Guerrero-Mandujano et al., 2017). OMVs are composed of OMs, containing LPS, phospholipids and OM proteins, and soluble periplasmic components like DNA and proteins. OMVs function in bacterial communication, cell wall remodeling, horizontal gene transfer, defense, and pathogenicity (Mashburn and Whiteley, 2005; Bonnington and Kuehn, 2016; Crispim et al., 2019); their composition may thus differ from that of OM and periplasm (Schwechheimer et al., 2013; Kim et al., 2018; Malge et al., 2018). The size of the vesicles depends on the given strain but generally ranges from 50 to 250 nm in diameter (Beveridge, 1999). Vesiculation is induced as response to different stress factors, like the presence of antimicrobial or toxic particles or unfavorable environmental conditions, for example, nutrient deficiency or elevated temperatures (Loeb, 1974; Manning and Kuehn, 2011; Schwechheimer et al., 2013; Bai et al., 2014). Host cell OMVs can bind and rapidly remove free phage particles from a solution and effectively reduce phage activity (Manning and Kuehn, 2011; Biller et al., 2014; Parent et al., 2014; Reyes-Robles et al., 2018). However, the mechanisms of this interaction, that is time-resolved observation of the all involved steps on a molecular level, have not been studied in detail so far.

In this work, we have analyzed *Salmonella* phage P22 in the presence of OMV prepared from its host, *Salmonella enterica* ssp. *enterica* sv. Typhimurium (S. Typhimurium). Podovirus P22 is an O-antigen-specific, transducing phage with a short, non-contractile tail. P22 is a well-established model system for studying DNA transduction, *Salmonella* genetics, and lysogeny (Prevelige, 2006; Casjens and Grose, 2016). P22 uses LPS as its receptor, and tailspike protein (TSP)-mediated cleavage of the O-antigen part then positions the phage particle on the cell surface, where the particle opens (Andres et al., 2010). *In vivo*, genome transfer is mediated by a set of P22 ejection proteins that contribute to forming a channel-like structure that provides access to the cytosol by bridging the cell wall (Wang et al., 2019). *In vitro*, purified protein-free LPS aggregates mimicking the OM were sufficient to elicit DNA release into the solution (Andres et al., 2010). We wanted to extend this *in vitro* system to a more complex receptor system and have analyzed dynamics of DNA release from phage P22 in the presence of OMVs. We used large-scale OMV preparations to obtain sufficient material for fluorescence spectroscopy studies (Thein et al., 2010). Our *in vitro* analyses show that OMVs efficiently rendered P22 phage particles non-infective. OMV-triggered P22 phages ejected their DNA more rapidly than LPS-triggered P22 phages. However, and in contrast to pure LPS preparations, when triggered by OMVs, only a subfraction of these phages also injected their DNA into the vesicles.

## Materials and Methods

### Materials and Bacterial Strains

Yo-Pro®-1 iodide was obtained from Invitrogen (Thermo Fisher Scientific Life Technologies, Darmstadt, Germany), and Purpald® (4-Amino-3-hydrazino-5-mercapto-1,2,4-triazole and 4-Amino-5-hydrazino-1,2,4-triazole-3-thiol) was obtained from Sigma Aldrich (Merck KGaA, Darmstadt, Germany). The standard buffer in all experiments was 10 mM Tris–HCl and 4 mM MgCl_2_, pH 7.6. *Salmonella* strains S. Typhimurium AroA::Tn10 iroBC::kan (LPS and vesicle preparation) and S. Typhimurium DB 7136 LT2 (phage propagation) were from our laboratory collections (Tindall et al., 2005). The hypervesiculating strain S. Typhimurium ATCC 14028 MvP2390 was provided by Prof. Dr. Michael Hensel (University Osnabrück). Preparation of LPS has been described previously (Andres et al., 2010). LPS was quantified in all OMV samples by the Purpald® assay, and the protein content of OMV was determined with bicinchoninic acid (BCA; Smith et al., 1985; Lee and Tsai, 1999). PEG 8000 was purchased from Roth (Carl Roth GmbH, Karlsruhe, Germany). All other chemicals used were of analytic grade and double-distilled water was used throughout.

### Preparation of Outer Membrane Vesicles From Cell Lysates

OMVs present after mechanical cell lysis were prepared according to Thein et al. (2010). Briefly, S. Typhimurium AroA::Tn10 iroBC::kan were grown in LB medium supplemented with 50 μg ml^−1^ kanamycin overnight at 37°C. After cell harvest, cells were incubated with 0.1 mg ml^−1^ lysozyme and 10 μg ml^−1^ DNase I on ice and lysed with French press. Membranes were collected by centrifugation at 75,400 × *g* for 30 min at 4°C and suspended in standard buffer. For OMV purification with sucrose density gradients (“OMV Suc”), total membranes were loaded on a three-step sucrose density gradient (3 ml 75% w/v, 5 ml 50% w/v, and 3 ml 25% w/v) and centrifuged at 250,000 × *g* for 12 to 16 h at 4°C to separate inner membrane (IM) and OM fractions. The OM accumulated at the 50/75% sucrose interface. The harvested OM fraction was diluted in water, collected at 30,000 × *g* (30 min) and stored at 4°C. Alternatively, OMVs were obtained by selective detergent solubilization (“OMV Det”). For this, the total membranes after French press lysis were incubated with 1% N-lauroylsarcosine and centrifuged at 30,000 × *g* for 30 min at 4°C to solubilize the IM. To wash OMV, they were suspended in water, collected at 30,000 × *g* (30 min) and stored at 4°C.

### Preparation of Outer Membrane Vesicles Naturally Budded Into *Salmonella* Culture Supernatants

Hypervesiculating S. Typhimurium ATCC 14028 MvP2390 were grown in LB medium supplemented with 50 μg ml^−1^ kanamycin at 37°C to an OD_600 nm_ of 1.5. Cells and cell debris were removed by two subsequent centrifugation steps at 4000 × *g* (15 min each, 4°C). The supernatant was filtered twice on Whatman cellulose acetate 0.45 μm and 0.20 μm (GE Healthcare Europe GmbH, Freiburg, Germany), and OMVs (“OMV budded”) were collected from the filtrate at 38,500 × *g* (3 h, 4°C) and stored in phosphate-buffered saline (PBS) at 4°C.

### Electron Microscopy

EM images were recorded on a JEOL JEM1400-Plus transmission electron microscope (TEM). Staining methods for Gram-negative bacteria samples have been described (Grin et al., 2011). Samples were loaded on Formvar-coated copper grids, stained with 1% (w/v) uranyl acetate and embedded in 1% (w/v) uranyl acetate and 0.4% (w/v) methyl cellulose.

### Interaction Studies of Phage Particles With LPS and OMVs

#### Plaque-Forming Assay

The plaque-forming assay monitors the decrease of infectious particles due to co-incubation with LPS or OMV. 5 × 10^3^ pfu ml^−1^ P22 bacteriophages were incubated with 2.5 μg ml^−1^ LPS or OMV LPS equivalents in standard buffer at 37°C. After different incubation times, 100 μl were taken, mixed with warm soft agar, and plated on thin LB agar plates. After overnight incubation at 37°C, the plaques were counted and plaque-forming units (pfu ml^−1^) were determined. As control, P22 bacteriophages were incubated with buffer only.

#### DNA Ejection Monitored by Yo-Pro Fluorescence

Bacteriophage P22 DNA ejection was followed by fluorescence spectroscopy as described before (Andres et al., 2010). OMVs for these experiments were prepared with the sucrose gradient method (OMV Suc). Briefly, 4 × 10^9^ pfu ml^−1^ P22 bacteriophages were incubated at 37°C with 10 μg ml^−1^ LPS or OMV LPS equivalents in the presence of 1.1 μM Yo-Pro®-1 iodide. The fluorescence signal was detected at 509 nm after excitation at 491 nm. After 170 min, DNase I was added to a final concentration of 10 μg ml^−1^. Curves were corrected for Yo-Pro phage staining in presence of O-antigen-depleted OMVs. Curves were fitted to a monoexponential function as described before (Andres et al., 2010; Chiaruttini et al., 2010).

$$F_{t}=F_{\max}•\left(1-e^{-k_{open}t}\right)$$ (1)

$F_{t}$ Fluorescence signal at time point t

$F_{\max}$ maximum signal amplitude

$k_{\mathrm{open}}$ reaction rate constant of phage particle opening

t time

For ejection experiments with PEG 8000, osmotic pressures at 37°C were calculated from the PEG weight fraction according to the empirical polynomial (Parsegian et al., 1986):

$$\Pi \left(atm\right)=-1.29\;G^{2}T+140G^{2}+4G$$

G Converted PEG weight percent (w) concentration: $G=\frac{w}{100-w}$

T Temperature (°C)

## Results

### Preparation and Characterization of S. Typhimurium Outer Membrane Vesicles

To investigate differences in preparation of OMVs and to study the effects of altered vesicle characteristics on bacteriophage interactions, different preparation protocols were employed (Table 1). Gram-negative bacteria shed OMVs in culture supernatants; however, the low concentrations usually require large preparation scales to obtain sufficient amounts for quantitative functional analyses. We therefore generated OMV samples by accumulation of OMs after mechanical lysis of S. Typhimurium cells. Either IMs and OMs were separated on sucrose density gradients or IMs were solubilized by detergents to collect the OM fraction by centrifugation (Thein et al., 2010). Moreover, and for comparison, we isolated OMVs from culture supernatant filtrates of the hypervesiculating S. Typhimurium strain MvP2390 that is defective in Braun’s lipoprotein (Dramsi et al., 2008).

**Table 1:** Preparation methods for OMVs from S. Typhimurium.

OMV sample^a^	Membrane preparation	Enzyme treatment	Separation of IM and OM	Vesicle diameter^b^	Protein/ LPS ratio^c^
OMV Det	Membrane accumulation	DNase I and lysozyme treatment	Solubilization of inner membrane	50–100 nm	0.178
OMV Det*	Membrane accumulation	---	Solubilization of inner membrane	50–425 nm	0.244
OMV Suc	Membrane accumulation	DNase I and lysozyme treatment	Sucrose density gradient	25–125 nm	0.174
Budded OMV	Naturally budded vesicles	---	---	25–100 nm	0.936

a Det, detergent method; Suc, sucrose gradient method.

b Determined by electron microscopy and dynamic light scattering (Supplementary Figure S1).

c BCA and Purpald assay.

**Figure 1:**
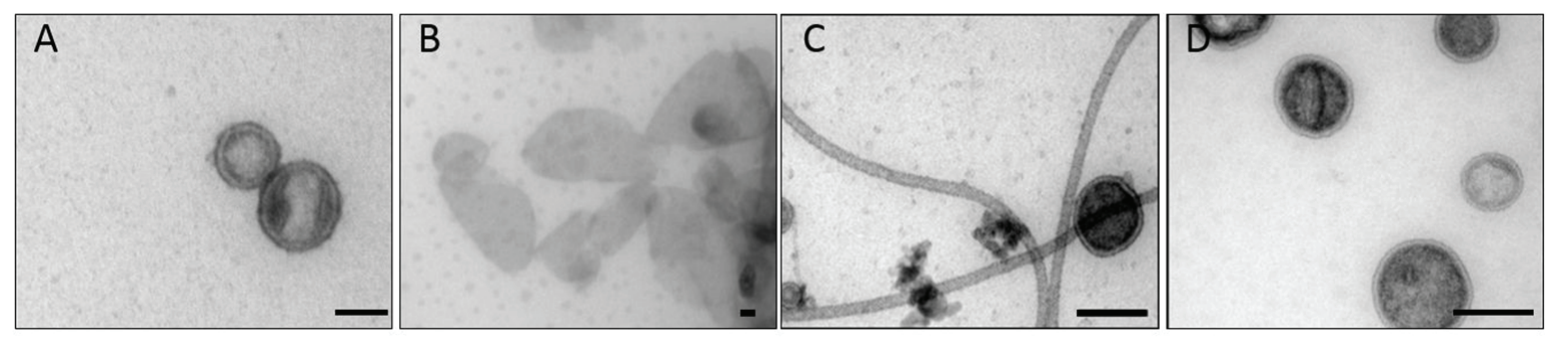
Transmission electron microscopy of *Salmonella* Typhimurium outer membrane vesicles (OMVs). **(A)** OMV Det, **(B)** OMV Det^*^, **(C)** budded OMV, **(D)** OMV Suc. The bar represents 50 nm.

Electron microscopy analysis showed that with all preparation methods, samples were obtained with spherical vesicles of 50–100 nm diameter (Figure 1). Stokes radii measured by dynamic light scattering confirmed these OMV size distributions (Supplementary Figure S1). To obtain homogeneously round-shaped vesicles from membrane accumulation, we noticed that a pre-lysis treatment of cells with DNase I and lysozyme was mandatory, because otherwise only large and irregular membrane aggregates were formed (Figure 1B and Supplementary Figure S2). The vesicles’ protein: LPS ratio was calculated from analyses with BCA and Purpald assays, respectively (Table 1). In contrast, IM fractions that could be isolated from sucrose gradients contained notably less vesicle-like structures (Supplementary Figure S3). OMVs from membrane accumulation contained typical OM markers, like OM proteins and LPS (Supplementary Figure S3). SDS-PAGE with glycan staining confirmed that they contained LPS with a typical O-antigen chain length distribution (Supplementary Figure S4). Hence, LPS in OMVs could be quantified with the Purpald assay. Furthermore, co-expression of a fluorescently labeled OmpA-mCherry construct as OM marker protein showed that it was located in the OMV fraction after membrane accumulation and sucrose density gradient separation (Supplementary Figure S3).

The budded OMVs from culture supernatants showed an increased protein content compared to OMVs collected by membrane accumulation and also a different protein composition as estimated from SDS-PAGE analysis (Supplementary Figure S5). As shown earlier, under typical growth conditions, naturally budded *Salmonella* OMVs contain an increased amount of cytosolic proteins (Bai et al., 2014), which may account for the elevated protein content. Moreover, EM images of our samples revealed flagella-like structures that apparently had co-purified with the OMVs. In principle, it would be also possible that additional periplasmic proteins are present in the lumen of these naturally budded vesicles or that they display increased amounts of OM proteins (Volgers et al., 2018). However, the small yield of these OMVs isolated from culture supernatants impeded a further detailed proteomic analysis.

### Interaction of Vesicles With Bacteriophage P22

**Figure 2:**
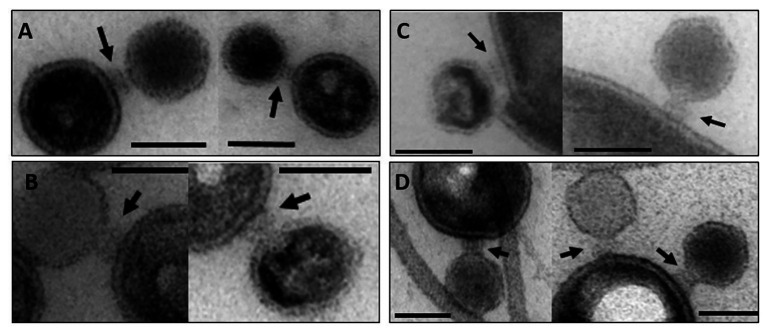
Transmission electron microscopy images of OMV incubated with bacteriophage P22. The arrows point to the phage’s baseplate. The bar represents 50 nm. **(A)** OMV Suc, **(B)** OMV Det, **(C)** OMV Det^*^, **(D)** budded OMV (*cf*. Table 1 for description of different OMV samples).

Transmission electron microscopy showed that P22 bacteriophages interact with OMVs. After overnight incubation at 37°C, the uranyl acetate stain reveals the OM structure as a bright ring enclosing the vesicle lumen that appeared in a darker, “negative” stain (Figure 2). All OMV preparations showed vesicles being bound by phages. Phages either showed intense staining of the capsid shell or a lighter appearance of their capsids, which we assign to DNA-ejected and non-ejected particles, respectively. Phages were attached to the vesicle surface with their tail apparatus (arrows in Figure 2), but at the given resolution, differences in the tail conformation of ejected and non-ejected phages cannot be verified. As a rough estimate from manual image inspection, we found about 25% of the vesicles with bound phage (Supplementary Figure S6). A quantitative analysis would require cryo-EM sample preparation and variation of the phage-OMV ratio to estimate proportions of bound and ejected versus bound and non-ejected phages (Parent et al., 2014; Reyes-Robles et al., 2018).

**Figure 3:**
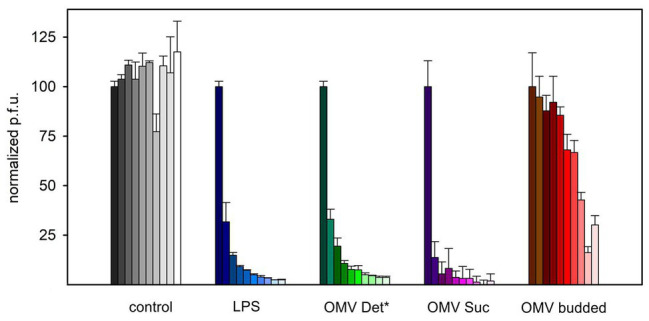
Reduction of P22 plaque formation on S. Typhimurium in the presence of LPS or OMV. 8 × 10^4^ P22 bacteriophages were incubated with 2.5 μg ml^−1^ LPS or 2.5 μg ml^−1^ OMV LPS equivalents at 37°C before analysis of plaque-forming units on S. Typhimurium. Increasing incubation times shown as fading colors: 0, 5, 10, 20, 30, 45, 60, 90, 120, 180, and 240 min. For characteristics of different OMV types *cf*. Table 1. Error bars represent results from three technical replicates.

To further validate irreversible binding and inactivation of phage P22 by OMVs, we analyzed P22-OMV mixtures for their ability to form plaques on the P22 S. Typhimurium host. With prolonged incubation times, we found that the plaque-forming units in the presence of membrane preparation-derived OMVs were reduced rapidly to less than 5% of the initial value (Figure 3). Interestingly, the inactivation of P22 by OMVs was well comparable to that found by a purified, protein-free LPS sample; accordingly, we assume that the proteins present in the OMVs did not influence the phage inactivation. Rather, as previously described, the isolated LPS receptor is a sufficient inhibitor to inactivate the O-antigen-specific P22 phage (Broeker and Barbirz, 2017; Broeker et al., 2019). However, whereas inactivation profiles for OMVs obtained from OM and LPS were highly similar, budded OMVs obtained from culture supernatants showed a much slower inactivation effect on phage P22, with about 25% of active phage particles remaining after 6 h of incubation. This might be related to their increased protein content, probably reducing the number and altering the distribution of surface-accessible LPS receptors, resulting in a slower decrease of infective, unbound phage particles. Flagella contaminations in this preparation might additionally have affected their capacity for phage inactivation.

### P22 Bacteriophage Particle Opening Analysis With Vesicles

Lysis and plaque-forming assays only show irreversible binding and inactivation of phage P22, but no information is obtained whether inactivated bacteriophage P22 particles have lost their DNA upon contact with OMVs. Moreover, from negative stain EM analyses, neither quantitative amounts of ejected phage nor dynamics of the DNA ejection process were accessible. We therefore monitored DNA ejection of bacteriophage P22 with a fluorescence ejection assay that has been described previously (Andres et al., 2010, 2012; Broeker et al., 2018, 2019). In this assay, purified host LPS is mixed with the bacteriophage. This triggers opening of the phage capsid and release of DNA in the surrounding solution that contains the fluorescent DNA-intercalating dye Yo-Pro. This leads to a rapid and intense fluorescence signal increase with increasing phage DNA concentrations in solution. In contrast, in the absence of LPS, phage particles remain intact and only show a weak background fluorescent signal, because the dye Yo-Pro has weak affinity to the highly condensed DNA inside the phage capsids. As a consequence, O-antigen-specific phages only show a DNA ejection signal when triggered with an intact LPS, whereas O-polysaccharide or lipid A alone or rough LPS do not elicit DNA release (Andres et al., 2010). For P22, as for other phages, monoexponential kinetic ejection profiles were measured with this method that describes the rate-limiting phage particle opening step (Chiaruttini et al., 2010; Andres et al., 2012).

**Figure 4:**
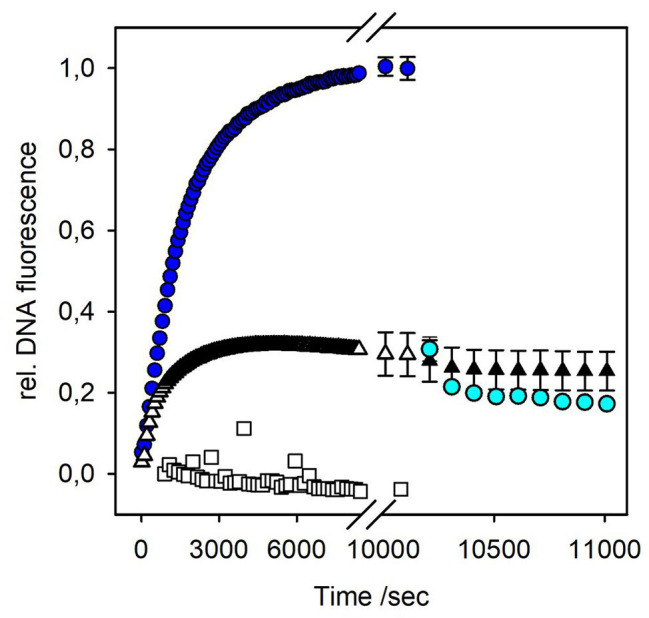
DNA release from bacteriophage P22 triggered by OMV or LPS. Incubation of 4 × 10^9^ pfu/ml P22 phages at 37°C in the presence of a fluorescent DNA-binding dye (1 μM Yo-Pro) with 10 μg ml^−1^ LPS (blue circles) or OMVs (white triangles; 10 μg ml^−1^ LPS equivalents, see Materials and Methods). Rate constants for phage particle opening were calculated from monoexponential fluorescence increase to *k_open,LPS_* = 5.89 ± 0.01 × 10^−4^ s^−1^, and *k_open,OMV_* = 10.00 ± 0.01 × 10^−4^ s^−1^, respectively (Chiaruttini et al., 2010). After 170 min, DNase I (10 μg ml^−1^) was added to the ejected phages, either LPS-triggered (cyan circles) or OMV-triggered (black triangles). Plot shows mean values of three technical replicates. For clarity, only every tenth point was plotted and exemplary error bars are given for the last points of the ejection curves and for data in presence of DNase I only. Plots with all error bars can be found in Supplementary Figure S9. As an O-antigen-free control, 10 μg ml^−1^ OMV LPS equivalents were incubated overnight with 10 μg ml^−1^ P22TSP at 37°C prior to the ejection experiment (white squares).

Co-incubation of P22 phage and OMVs in the presence of Yo-Pro resulted in a monoexponential increase of the fluorescence signal, reaching a plateau after about 170 min (Figure 4). Neither OMVs alone nor a control in which phage was incubated with OMVs that lack the phage O-antigen receptor showed a notable fluorescence signal increase. Hence, the fluorescence increase in the presence of OMVs monitors phage DNA liberated from the phage capsids. We monitored similar DNA release profiles from phage P22 with all OMVs irrespective of their source and purification protocol (Supplementary Figure S7). However, the naturally budded OMVs contained protein impurities and were available only at an overall poor yield (see above). In all fluorescence experiments, we therefore worked with OMVs obtained from accumulated membranes with the sucrose gradient method (*cf*. Materials and Methods).

To check whether phages ejected their DNA into the vesicles or into the solution, we added DNase I after the ejection signal had reached a plateau. If DNA is free in solution, nucleotides produced by DNase I will no longer intercalate with Yo-Pro, resulting in a decrease of the fluorescence signal (Andres et al., 2010). However, the fluorescence signal obtained from P22 DNA ejection in the presence of OMVs did not decrease (Figure 4). Apparently, when triggered by OMVs, all DNA ejected from phage was not accessible to DNase I, because it was protected either inside the vesicles or inside the phage capsids.

The plaque-forming assay showed that OMVs can rapidly inactivate phage particles in solution. To check whether all phages in the fluorescence experiment were associated with OMVs, we added free LPS to the mixtures 170 min after the reaction was started. If at this time point a significant amount of phage particles had remained free in solution, a signal increase due to DNA release upon phage interactions with the newly added free LPS receptor should be observed. However, we did not observe any further signal increase upon LPS addition (Supplementary Figure S8). This result is in agreement with the plaque-forming assay where OMV rapidly cleared off phage particles from the solution. We therefore conclude that all P22 phage particles in the fluorescent assay were rapidly and irreversibly associated with OMVs and could no longer interact with newly added LPS.

In the fluorescence assay, the OMV concentration was calculated as LPS units determined with the Purpald test to be able to compare OMV-triggered phage ejection profiles with those triggered with purified LPS. Comparison of kinetic constants for DNA ejection triggered either by LPS or OMVs showed that particle opening velocities with OMVs were increased about 2-fold relative to LPS (Figure 4). However, only about 30% of the signal amplitude were reached compared to phages triggered with a pure LPS sample. Even if we increased OMV concentrations 3-fold, we did not observe an increase in the amount of ejected DNA (Supplementary Figure S8). This means that only a part of the phage particles have lost their DNA in the presence of OMVs, in contrast to purified LPS, in presence of which all particles completely exposed their DNA to the solution (Andres et al., 2010).

OMVs used in the fluorescence experiments were prepared from accumulated OMs after French press cell lysis. We cannot fully exclude the idea that a part of the OMVs obtained by this method might be inverse, i.e., with the LPS at the inside and the phospholipid part at the outside. O-antigen-specific phage P22 would not bind to a vesicle with O-antigen chains on the inside and not eject its DNA and would thus remain inert against the inside-out vesicle fraction. OMVs in this study were quantified *via* their LPS concentration. We did not find an increase in DNA ejection when we increased the OMV concentration from 10 μg ml^−1^ LPS to 30 μg ml^−1^ LPS (Supplementary Figure S8). This means that at 10 μg ml^−1^ LPS OMV concentration, all phage particles in the system can interact with the OMVs. If a part of the vesicles were inside out, we should observe an increase in ejected phages because with increased OMV concentration, more phage O-antigen receptors become exposed. Moreover, we enzymatically removed the O-antigen from the vesicle surface by incubation with purified P22 tailspike protein prior to exposing them to phage P22. The resulting O-antigen-depleted vesicles could not trigger DNA ejection from phage P22 (Supplementary Figure S8). From these controls, we conclude that the fluorescence signal observed in our experiments is exclusively related to a phage interaction with vesicles that expose LPS on the outside.

### DNA Ejection in the Presence of Polyethylene Glycol (PEG)

Protein-free, highly pure LPS preparations that are present as aggregates free in solution could trigger particle opening in all P22 phages present in a reaction. However, for P22 DNA ejection triggered by OMVs, only about 30% of DNA signal was obtained. Moreover, this signal was not accessible to DNase I. These findings imply two scenarios for P22 DNA ejection in the presence of OMV, either (i) 30% of the particles have completely ejected their DNA, whereas for the remaining fraction no DNA release occurs, or (ii) all particles have released their DNA, but only to about 30%. To distinguish between these two situations, we analyzed DNA release from phage P22 in the presence of increasing PEG concentrations. It has been shown previously for siphovirus λ that DNA ejection is driven by the intra-capsid osmotic pressure and that at increasing osmotic pressures, less DNA could leave the λ capsids (Evilevitch et al., 2003, 2005). Also for P22, it was shown that at increasing osmotic pressures and when triggered with LPS and OM proteins, part of its DNA remained in the phage capsid and was protected from DNase cleavage (Jin et al., 2015).

**Figure 5:**
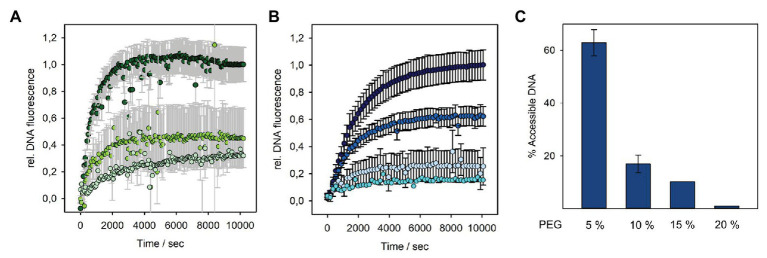
DNA release from bacteriophage P22 triggered by OMV or LPS at different solution osmotic pressures. Incubation of 4 × 10^9^ pfu/ml P22 phages at 37°C in the presence of a fluorescent DNA-binding dye (1.1 μM Yo-Pro) and increasing PEG 8000 concentrations with **(A)** OMV (10 μg ml^−1^ LPS equivalents) at 0% PEG (dark green, 0 atm), 15% PEG (grass green, 3.58 atm), or 20% PEG (pale green, 6.77 atm) or **(B)** 10 μg ml^−1^ LPS at 0% PEG (dark blue, 0 atm), 5% PEG (medium blue, 0.46 atm), 15% PEG (light blue, 3.58 atm), or 20% PEG (cyan, 6.77 atm; in brackets: osmotic pressure of the solution at 37°C, see Materials and Methods). Plots with error bars show mean values of three technical replicates. Values at 20% PEG are estimates from single measurements. Only every 10th data point was plotted. LPS curves in **(B)** had notably smaller error bars (black) than the OMV curves in (**A**; error bars plotted in gray for clarity). **(C)** DNA remaining accessible to DNase I after LPS-triggered DNA release from phage P22 at different PEG concentrations. Percentage values after DNase I incubation were calculated relative to the maximal DNase accessible fluorescence amplitude at 0% PEG (see Supplementary Figure S10 for the measured fluorescence signals and ejection curves at more PEG concentrations).

We therefore analyzed the DNA fluorescence increase in the presence of different concentrations of PEG 8000, when P22 was mixed with OMVs (Figure 5A). At 15% (3.58 atm) PEG, we obtained about 40% of the initial signal. This shows that an external osmotic pressure could effectively reduce DNA ejection from vesicle-associated phages. However, earlier work showed that, at this osmotic pressure (3.58 atm), still around 70% of the P22 DNA could leave the capsid when triggered with LPS or a mixture of LPS and the outer membrane protein OmpA (Jin et al., 2015). Hence, P22 triggered by OMVs ejected less DNA at increased external osmotic pressures, illustrating that OMVs represent an OM receptor system different from pure LPS or OM protein–LPS mixtures. Furthermore, the notable effect also of lower PEG concentrations on DNA release confirms that only about 30% of the P22 particles are actively ejecting their DNA in the presence of OMVs. At 5% PEG (0.46 atm), we already observed a signal decrease to 70% (Supplementary Figure S10). If all particles in the system were actively releasing only a part of their DNA, these low osmotic pressures would not elicit a pronounced effect on ejection because the initial release of only a small fraction of DNA is also possible against elevated osmotic pressures.

It was shown for other systems that at higher PEG concentrations, the DNA ejection can be completely stopped (Evilevitch et al., 2003; Jin et al., 2015). However, in our fluorescence setup, we could not further increase PEG concentrations, as this resulted in high signal-to-noise ratio and large standard deviations. This was probably due to vesicle solubility hampered by PEG or slow equilibration of the mixtures in the viscous solution. At an osmotic pressure of 6.77 atm (20% PEG), we can thus only estimate that P22 was able to still release about 20% of its DNA. Moreover, and as described above, also in the presence of PEG, it was not possible to notably reduce the DNA signal by the addition of DNase. Again, we assume that the DNA was injected into the vesicle lumen, impeding quantification of DNA that had remained in the phage capsid at the given PEG concentration by DNA accessibility.

As DNA protection from DNase inside the P22 capsid upon ejection against increasing osmotic pressures had only been accessed on agarose gels before (Jin et al., 2015), we also analyzed purely LPS-triggered DNA release in the presence of PEG with time-resolved Yo-Pro fluorescence (Figure 5). Interestingly, we found that much lower osmotic pressures could completely halt DNA ejection from phage P22 than it was reported in the previous work. Already at 15% PEG (3.58 atm), nearly no free DNA was detectable in the solution and was protected from DNase cleavage, indicating that DNA release from phage P22 had come to a nearly complete stop. We assume that this different result might be due to our different LPS preparation method that ensured the complete absence of even minute contaminations of other OM components like proteins or phospholipids.

In summary, our results confirmed that irrespectively of the triggering system, either OMVs or LPS, particle opening and DNA egress from P22 are driven by osmotic pressure inside the capsid. Moreover, whereas with LPS, 100% of the phages can eject, with OMV, an inert fraction of about 70% remains, which is unable to liberate its DNA.

## Discussion

Host infection of O-antigen-specific bacteriophages is initiated by the binding of phage particles to LPS. Degradation or deacetylation of the polysaccharide anchors the phage irreversibly to the cell surface, presumably in a defined distance to ensure proper spatial positioning for building a membrane-spanning infection apparatus (Andres et al., 2010; Broeker and Barbirz, 2017; Prokhorov et al., 2017; Wang et al., 2019). It is characteristic for O-antigen-specific phages that *in vitro* preparations of LPS render them non-infectious because they elicit particle opening and DNA release (Andres et al., 2012; Broeker et al., 2018, 2019). In contrast, neither O-polysaccharide alone nor lipid A or rough-type LPS with short O-antigen chains is sufficient to trigger particle opening. Apparently, the membrane-like character of LPS preparations that form multilamellar aggregates in solution is a prerequisite to trigger irreversible rearrangements in the phage tail apparatus, ultimately leading to DNA ejection (Richter et al., 2011). However, to also release P22’s ejection proteins, the presence of OM proteins is necessary, illustrating that LPS-triggered DNA release only covers a subset of the initial phage infection steps (Parent et al., 2014). In this work, we have investigated the interactions of the O-antigen-specific bacteriophage P22 with OMVs that present LPS and other OM components in a defined way to the phage.

### OMV Composition and Structure Is Highly Dependent on the Formation Process

OMVs are small vesicles with the same membrane organization as found in the Gram-negative OM, i.e., an inner leaflet of phospholipids and an outer LPS layer (Whitfield and Trent, 2014). OMVs thus resemble the OM to a much higher extent as compared to pure LPS aggregates. From this, it naturally follows that vesicles can be active in controlling bacteriophages in a bacterial population, and although these interactions have been unambiguously observed before with electron microscopy, they have so far only been addressed by a few studies (Manning and Kuehn, 2011; Biller et al., 2014; Parent et al., 2014; Reyes-Robles et al., 2018). One major drawback is the difficulty to obtain enough OMV material from culture supernatants (Thein et al., 2010). We therefore used OM fractions where the vesicles were formed during cell lysis. As a matter of fact, OMV composition, especially with respect to the protein content, is highly dependent on the conditions under which the vesicles are formed. During a natural budding event, bacterial cells control the OMV protein load, for example in response to nutrient availability in the medium or to distribute antibiotic resistance factors like β-lactamases (Bai et al., 2014; Kim et al., 2018). Moreover, the size of budded vesicles is dictated by the strain of origin, formation mechanism, and LPS and protein composition (Deatherage et al., 2009; Kulp and Kuehn, 2010; Schwechheimer and Kuehn, 2015; Toyofuku et al., 2019).

We have used different techniques to obtain vesicles from OM preparations. Here, the formation of vesicles from the enriched OM is driven by hydrophobic effects and leaflet asymmetry (Park et al., 2015; Rozycki and Lipowsky, 2015). We found that DNaseI and lysozyme treatment were important prerequisites to obtain OMVs with size and shape similar to naturally budded OMVs. This emphasizes that inefficient separation of IMs and OMs most probably resulted in contamination with IM components. In the accumulated OM fraction this hence changed membrane thickness and resulted in large and irregular vesicle size. Moreover, the separation method, i.e., either sucrose gradient or detergent treatment, can influence the extent of residual IM contaminations in OMVs, as well as the protein content (Thein et al., 2010). Accordingly, we observed differences in the protein pattern in OMVs prepared *via* sucrose gradient or with detergent, although the total protein content was approximately the same. All quantitative bacteriophage-OMV interaction fluorescence analyses in this work were thus carried out with OMVs prepared by a single technique, i.e., the sucrose gradient method. We are aware that OMVs obtained from different preparation methods might show different results. Even more, to further address OMV function in bacterial control of bacteriophage populations, naturally budded vesicles should be tested, for which large-scale preparations have also been reported (Hsia et al., 2016). Nevertheless, we claim that our reductionist *in vitro* system can add insight into bacteriophage interactions with their O-antigen receptors when part of a more complex membrane system as present in OMVs.

### Bacteriophage P22 OMV Interactions Clearly Differ From Interactions With Isolated LPS Receptors

OMVs produced from bacteriophage P22’s S. Typhimurium host act as a decoy for phage particles. Like free LPS aggregates, incubation with OMV preparations rapidly reduced the number of infective particles in the solution. Phages that are in contact with OMVs in the majority were not primed for genome transfer. Rather, they were inactivated, mostly by simply binding and blocking the phage on the OMV by a so far unknown mechanism and, to a smaller extent, by genome loss as shown in this and other studies. Differences in the decrease of infective particle numbers were found with OMVs from different preparations. In particular, the naturally budded OMVs isolated from culture supernatants reduced infective P22 particle numbers to a lesser extent compared to OMVs prepared from OM fractions. It is important to note here that in the experiment, all OMV samples were adjusted to the same LPS content. However, lacking proteome characterization of budded OMVs, we can only speculate whether they contained a different OM protein decoration that interfered with LPS receptor accessibility or influenced the membrane curvature. Moreover, additional periplasmic proteins inside the OMVs could have slowed down P22 inactivation, for example, by increasing the osmotic pressure in the vesicle lumen. Also, if these OMVs presented less LPS on their surface, TSP cleavage of the O-antigen receptor might compete with irreversible phage attachment (Broeker et al., 2019) and prevent phage inactivation. During P22 isolation, OMVs were not pulled down together with the phage, in contrast to podovirus Sf6 that co-purified with OMVs through interactions with OmpA and OmpC (Parent et al., 2014).

*In vitro* fluorescence analyses have been used regularly to monitor DNA ejection from phages triggered with their isolated receptors, either OM proteins or LPS, in bulk or on a single particle level (Mangenot et al., 2005; Andres et al., 2010, 2012; Chiaruttini et al., 2010; Gonzalez-Garcia et al., 2015; Broeker et al., 2019). In these experimental setups, a DNA-specific dye fluorescence increase is observed due to DNA release from a capsid-confined state into a more relaxed state in the solution. For P22 it was shown that particles open and eject about 80% of their DNA content when triggered with LPS (Andres et al., 2010). The kinetic profile of this process is conserved in other podovirus systems analyzed with the same method, but different LPS receptors (Broeker et al., 2018). This reflects that the rate-limiting particle opening step most probably involves rearrangements in conserved parts of the podovirus tail assembly.

In this work, we have now used OMVs to trigger *in vitro* particle opening of bacteriophage P22 and could monitor time-resolved DNA release with a DNA-sensitive fluorescence stain. This is in contrast to cryo-EM analyses that compared end points of ejection by quantifying phages that had lost their DNA (Reyes-Robles et al., 2018). Phage-released DNA in the presence of OMVs was fully protected from DNase degradation, in contrast to the DNA released with pure LPS in solution. This indicates that OMVs and the phage were intimately linked in a stable complex where DNA ejection was exclusively targeted to the vesicle lumen. Similarly, EM images of bacteriophage-bound OMVs of the marine cyanobacterium *Prochlorococcus* suggested that DNA transfer into the vesicle lumen had taken place, as the tail of the myovirus was contracted and the capsid was empty (Biller et al., 2014). Our fluorescence bulk kinetic study typically monitored approximately 10^9^ P22 particles, of which only about 30% ejected their DNA upon contact with OMVs. Also for other phages, cryo-EM imaging and quantification showed that a majority of particles had retained their DNA inside the capsid when bound to OMVs, for example, *Vibrio cholerae* phages in up to 90% of all particles imaged (Reyes-Robles et al., 2018). This fraction is similar to what was found with cryo-EM for OMV-bound *Shigella* podovirus Sf6 (Parent et al., 2014). So far, we have failed with attempts to physically separate the OMV-phage P22 complexes to individually analyze the vesicles’ DNA content after phage contact. Similar problems had also been reported before for OMVs binding to bacteriophage T4 (Manning and Kuehn, 2011). Apparently, it is difficult to find solubilization conditions that either address the OMV or the phage without affecting the integrity of the other. Moreover, irreversible phage attachment to OMVs might already destabilize the phage even if it has not released its DNA.

Our experiments furthermore showed that, compared to using pure LPS as receptor for triggering DNA release from phage P22, the fluorescence signal obtained with OMV receptors went into saturation twice as fast. In OMVs, P22 now encountered a more complex membrane system compared to LPS. LPS has a unique solution behavior, forming multilamellar aggregates that presumably mimic membrane-like structures addressed by the phage *in vitro*, but do not represent all characteristics of the OM heterobilayer (Andres et al., 2010; Richter et al., 2011). In OMVs in contrast, OM proteins are present that might in a so far unknown way accelerate the phage particle opening step and the subsequent DNA release. This becomes evident especially when analyzing P22 at elevated osmotic pressures. In this work, we showed that DNA release triggered by highly pure LPS lacking OM proteins already ceased at external osmotic pressures of about 3.6 atm. However, it has been shown earlier that in the presence of OM proteins at this osmotic pressure, P22 still released a fraction of its DNA (Jin et al., 2015). Moreover, OM proteins enabled the release of P22’s ejection proteins, in contrast to LPS alone. Our actual DNA ejection studies are in line with these findings. P22 in the presence of OMVs at 6.8 atm osmotic pressure still showed partial DNA loss, emphasizing that progression of DNA release from the phage particle is intimately coupled to the receptor type. For P22, it was shown that this can be pure LPS aggregates, a combination of LPS and OM proteins or OMVs. We note that in comparison of the present study with the one cited above (Parent et al., 2014), the latter showed no difference in fractional DNA release between samples triggered with LPS alone or with a mixture of OmpA and LPS. As a reason for this ambiguity, we assume that different LPS preparation protocols may have resulted in incomplete OM protein removal. In summary, the new results emphasize the critical role of OM composition for phage control. Bacteria might exploit this by adjusting their OMV contents in response to phage attack.

Our *in vitro* experiments again illustrate that all energy needed for DNA release from bacteriophages is already stored in the system (Keller et al., 2017). The phage genome is highly confined inside the capsid, packaged against the DNA intramolecular electrostatic repulsion forces by an ATP-hydrolysis-driven motor (Smith et al., 2001). Accordingly, increasing external osmotic pressures can counteract genome liberation (Evilevitch et al., 2003). For siphovirus λ with a long, non-contractile tail, an internal capsid pressure of about 20 atm was estimated that was dependent on the relationship of genome length and capsid size (Grayson et al., 2006). However, external factors were shown to significantly influence the genome ejection characteristics of λ, illustrating the complex behavior of the DNA polyelectrolyte both inside the capsid and when transferred into the solution (Evilevitch, 2018). Also, the osmotic status of OMVs interacting with phage P22 might play a role in the observed DNA release process. If OMVs had a significant turgor, for example, due to their luminal protein content, the amount of DNA release from P22 would be insensitive to low osmotic pressures in the surrounding solution. However, also with OMVs, we observed that the amount of ejected DNA decreased with increasing PEG solution concentrations. This indicates (i) that presumably no osmotic pressure existed in the OMVs and (ii) that ejecting P22 particles could transfer substantial amounts of DNA inside the vesicles. As already discussed above, we could not directly quantify how much DNA remained inside the phage capsid and how much was translocated into the vesicle lumen. Nevertheless, the notable effect of low PEG concentrations on the ejection signal shows that more than 30% of the DNA must have left the capsid on the level of an individually ejecting phage, in agreement with cryo-EM studies with other phage systems (Parent et al., 2014; Reyes-Robles et al., 2018). Hence, the reduced signal amplitude observed in fluorescence curves obtained with OMVs is due to a reduced number of particles actively ejecting when compared to particles triggered with free LPS. In the future, this bulk study-derived hypothesis needs further evaluation to understand molecular details of OMV-mediated bacteriophage inhibition.

## Conclusion

Bacteriophage P22 *in vitro* particle opening studies with OMVs revealed that the action of the LPS receptor on the phage is modulated when LPS is part of a more complex OM system. Although OMVs could also effectively reduce the number of infective particles, inactivation was not driven by DNA release from all particles as observed with pure LPS fragments. Rather, a remarkable amount of phages did not eject their DNA. The mechanism of this OMV-driven bacteriophage inactivation remains to be elucidated and may be steered by different OMV properties. For example, vesicular curvature and protein decoration result in enhanced membrane tension and variable membrane thickness, respectively (Wu et al., 2014; Huang et al., 2017). Also, the presence of OM proteins as co-receptors influences the phage interactions (Parent et al., 2014). Moreover, multiple binding of P22 phages on small vesicles may induce mutual obstruction between the phages, prohibiting the DNA ejection. Importantly, we found that a preparation of naturally budded vesicles showed a much smaller capacity to inhibit the phage compared to the fraction obtained during mechanical cell lysis. This emphasizes that vesicle composition is a major control point for their function as phage blocking agents. From a more general point of view, if bacteria shed OMVs to block a phage population, they might employ a mechanism to inhibit substantial DNA liberation into the OMVs. As OMVs may also act in gene transfer, bacteria thus avoid another route with which phage genes could enter the bacterial cell. Further analyses will elucidate how the biochemical composition and properties of OMVs are linked to bacteriophage population regulation in a given functional context.

## Data Availability Statement

All datasets generated for this study are included in the article/Supplementary Material.

## Author Contributions

SB, DL, NR, and NB conceptualized the study and methodology. MS, NR, and AS performed the experiments. MS, NR, NB, and SB evaluated and visualized the data. MS, SB, DL, and AS wrote the manuscript. SB, DL, AS, NR, and NB supervised the study. SB and DL acquired funding. All authors contributed to the article and approved the submitted version.

### Conflict of Interest

The authors declare that the research was conducted in the absence of any commercial or financial relationships that could be construed as a potential conflict of interest.

## Supplementary Material

The Supplementary Material for this article can be found online at:

https://www.frontiersin.org/articles/10.3389/fmicb.2020.510638/full#supplementary-material

OMV size distributions from dynamic light scattering (Supplementary Figure S1), additional TEM images (Supplementary Figures S2, S3, and S6) and characterization of OMVs (Supplementary Figures S3–S5) as well as additional DNA ejection curves (Supplementary Figures S7–S9) can be found in the Supplementary Data Sheet 1.

## References

[ref2] Andres D. Hanke C. Baxa U. Seul A. t. Barbirz S. Seckler R. (2010). Tailspike interactions with lipopolysaccharide effect DNA ejection from phage P22 particles in vitro. J. Biol. Chem. 285, 36768–36775. 10.1074/jbc.M110.169003, PMID: 20817910 PMC2978605

[ref3] Andres D. Roske Y. Doering C. Heinemann U. Seckler R. Barbirz S. (2012). Tail morphology controls DNA release in two Salmonella phages with one lipopolysaccharide receptor recognition system. Mol. Microbiol. 83, 1244–1253. 10.1111/j.1365-2958.2012.08006.x, PMID: 22364412

[ref4] Bai J. Kim S. I. Ryu S. Yoon H. (2014). Identification and characterization of outer membrane vesicle-associated proteins in *Salmonella enterica* serovar Typhimurium. Infect. Immun. 82, 4001–4010. 10.1128/Iai.01416-13, PMID: 24935973 PMC4187864

[ref5] Beveridge T. J. (1999). Structures of gram-negative cell walls and their derived membrane vesicles. J. Bacteriol. 181, 4725–4733. 10.1128/JB.181.16.4725-4733.1999, PMID: 10438737 PMC93954

[ref6] Biller S. J. Schubotz F. Roggensack S. E. Thompson A. W. Summons R. E. Chisholm S. W. (2014). Bacterial vesicles in marine ecosystems. Science 343, 183–186. 10.1126/science.1243457, PMID: 24408433

[ref7] Bonnington K. E. Kuehn M. J. (2016). Outer membrane vesicle production facilitates LPS Remodeling and outer membrane maintenance in Salmonella during environmental transitions. mBio 7:e01532–16. 10.1128/mBio.01532-16, PMID: 27795394 PMC5082901

[ref8] Broeker N. K. Barbirz S. (2017). Not a barrier but a key: how bacteriophages exploit host’s O-antigen as an essential receptor to initiate infection. Mol. Microbiol. 105, 353–357. 10.1111/mmi.13729, PMID: 28618013

[ref9] Broeker N. K. Kiele F. Casjens S. R. Gilcrease E. B. Thalhammer A. Koetz J.. (2018). In vitro studies of lipopolysaccharide-mediated DNA release of podovirus HK620. Viruses 10:289. 10.3390/v10060289, PMID: 29843473 PMC6024685

[ref10] Broeker N. K. Roske Y. Valleriani A. Stephan M. S. Andres D. Koetz J.. (2019). Time-resolved DNA release from an O-antigen-specific Salmonella bacteriophage with a contractile tail. J. Biol. Chem. 294, 11751–11761. 10.1074/jbc.RA119.008133, PMID: 31189652 PMC6682738

[ref11] Casjens S. R. Grose J. H. (2016). Contributions of P2-and P22-like prophages to understanding the enormous diversity and abundance of tailed bacteriophages. Virology 496, 255–276. 10.1016/j.virol.2016.05.022, PMID: 27372181 PMC4969182

[ref12] Celluzzi A. Masotti A. (2016). How our other genome controls our Epi-genome. Trends Microbiol. 24, 777–787. 10.1016/j.tim.2016.05.005, PMID: 27289569

[ref13] Cenens W. Makumi A. Govers S. K. Lavigne R. Aertsen A. (2015). Viral transmission dynamics at single-cell resolution reveal transiently immune subpopulations caused by a carrier state association. PLoS Genet. 11:e1005770. 10.1371/journal.pgen.1005770, PMID: 26720743 PMC4697819

[ref14] Chiaruttini N. de Frutos M. Augarde E. Boulanger P. Letellier L. Viasnoff V. (2010). Is the in vitro ejection of bacteriophage DNA quasistatic? A bulk to single virus study. Biophys. J. 99, 447–455. 10.1016/j.bpj.2010.04.048, PMID: 20643062 PMC2905079

[ref15] Crispim J. S. Dias R. S. Laguardia C. N. Araujo L. C. da Silva J. D. Vidigal P. M. P.. (2019). Desulfovibrio alaskensis prophages and their possible involvement in the horizontal transfer of genes by outer membrane vesicles. Gene 703, 50–57. 10.1016/j.gene.2019.04.016, PMID: 30965126

[ref16] Deatherage B. L. Lara J. C. Bergsbaken T. Barrett S. L. R. Lara S. Cookson B. T. (2009). Biogenesis of bacterial membrane vesicles. Mol. Microbiol. 72, 1395–1407. 10.1111/j.1365-2958.2009.06731.x, PMID: 19432795 PMC2745257

[ref17] Doron S. Melamed S. Ofir G. Leavitt A. Lopatina A. Keren M.. (2018). Systematic discovery of antiphage defense systems in the microbial pangenome. Science 359:eaar4120. 10.1126/science.aar4120, PMID: 29371424 PMC6387622

[ref18] Dramsi S. Magnet S. Davison S. Arthur M. (2008). Covalent attachment of proteins to peptidoglycan. FEMS Microbiol. Rev. 32, 307–320. 10.1111/j.1574-6976.2008.00102.x, PMID: 18266854

[ref19] Erez Z. Steinberger-Levy I. Shamir M. Doron S. Stokar-Avihail A. Peleg Y.. (2017). Communication between viruses guides lysis-lysogeny decisions. Nature 541, 488–493. 10.1038/nature21049, PMID: 28099413 PMC5378303

[ref20] Evilevitch A. (2018). The mobility of packaged phage genome controls ejection dynamics. elife 7:e37345. 10.7554/eLife.37345, PMID: 30178745 PMC6122950

[ref21] Evilevitch A. Gober J. W. Phillips M. Knobler C. M. Gelbart W. M. (2005). Measurements of DNA lengths remaining in a viral capsid after osmotically suppressed partial ejection. Biophys. J. 88, 751–756. 10.1529/biophysj.104.045088, PMID: 15489301 PMC1305050

[ref22] Evilevitch A. Lavelle L. Knobler C. M. Raspaud E. Gelbart W. M. (2003). Osmotic pressure inhibition of DNA ejection from phage. Proc. Natl. Acad. Sci. U. S. A. 100, 9292–9295. 10.1073/pnas.1233721100, PMID: 12881484 PMC170911

[ref23] Feiner R. Argov T. Rabinovich L. Sigal N. Borovok I. Herskovits A. A. (2015). A new perspective on lysogeny: prophages as active regulatory switches of bacteria. Nat. Rev. Microbiol. 13, 641–650. 10.1038/nrmicro3527, PMID: 26373372

[ref24] Gonzalez-Garcia V. A. Pulido-Cid M. Garcia-Doval C. Bocanegra R. van Raaij M. J. Martin-Benito J.. (2015). Conformational changes leading to T7 DNA delivery upon interaction with the bacterial receptor. J. Biol. Chem. 290, 10038–10044. 10.1074/jbc.M114.614222, PMID: 25697363 PMC4400320

[ref25] Grayson P. Evilevitch A. Inamdar M. M. Purohit P. K. Gelbart W. M. Knobler C. M.. (2006). The effect of genome length on ejection forces in bacteriophage lambda. Virology 348, 430–436. 10.1016/j.virol.2006.01.003, PMID: 16469346 PMC3178461

[ref26] Grin I. Schwarz H. Linke D. (2011). Electron microscopy techniques to study bacterial adhesion. Adv. Exp. Med. Biol. 715, 257–269. 10.1007/978-94-007-0940-9_16, PMID: 21557069

[ref27] Guerrero-Mandujano A. Hernandez-Cortez C. Ibarra J. A. Castro-Escarpulli G. (2017). The outer membrane vesicles: secretion system type zero. Traffic 18, 425–432. 10.1111/tra.12488, PMID: 28421662

[ref28] Hsia C. Y. Chen L. X. Singh R. R. DeLisa M. P. Daniel S. (2016). A molecularly complete planar bacterial outer membrane platform. Sci. Rep. 6:32715. 10.1038/srep32715, PMID: 27600663 PMC5013322

[ref29] Huang C. J. Quinn D. Sadovsky Y. Suresh S. Hsia K. J. (2017). Formation and size distribution of self-assembled vesicles. Proc. Natl. Acad. Sci. U. S. A. 114, 2910–2915. 10.1073/pnas.1702065114, PMID: 28265065 PMC5358381

[ref30] Jin Y. Sdao S. M. Dover J. A. Porcek N. B. Knobler C. M. Gelbart W. M.. (2015). Bacteriophage P22 ejects all of its internal proteins before its genome. Virology 485, 128–134. 10.1016/j.virol.2015.07.006, PMID: 26245366 PMC4619139

[ref31] Keller N. Berndsen Z. T. Jardine P. J. Smith D. E. (2017). Experimental comparison of forces resisting viral DNA packaging and driving DNA ejection. Phys. Rev. E 95:052408. 10.1103/PhysRevE.95.052408, PMID: 28618627 PMC5953208

[ref32] Kim S. W. Park S. B. Im S. P. Lee J. S. Jung J. W. Gong T. W.. (2018). Outer membrane vesicles from beta-lactam-resistant *Escherichia coli* enable the survival of beta-lactamsusceptible *E. coli* in the presence of beta-lactam antibiotics. Sci. Rep. 8:5402. 10.1038/s41598-018-23656-0, PMID: 29599474 PMC5876404

[ref33] Kintz E. Davies M. R. Hammarlof D. L. Canals R. Hinton J. C. D. van der Woude M. W. (2015). A BTP1 prophage gene present in invasive non-typhoidal Salmonella determines composition and length of the O-antigen of the lipopolysaccharide. Mol. Microbiol. 96, 263–275. 10.1111/mmi.12933, PMID: 25586744 PMC4413052

[ref34] Kulp A. Kuehn M. J. (2010). Biological functions and biogenesis of secreted bacterial outer membrane vesicles. Annu. Rev. Biochem. 64, 163–184. 10.1146/annurev.micro.091208.073413, PMID: 20825345 PMC3525469

[ref35] Latka A. Maciejewska B. Majkowska-Skrobek G. Briers Y. Drulis-Kawa Z. (2017). Bacteriophage-encoded virion-associated enzymes to overcome the carbohydrate barriers during the infection process. Appl. Microbiol. Biotechnol. 101, 3103–3119. 10.1007/s00253-017-8224-6, PMID: 28337580 PMC5380687

[ref36] Leavitt J. C. Gogokhia L. Gilcrease E. B. Bhardwaj A. Cingolani G. Casjens S. R. (2013). The tip of the tail needle affects the rate of DNA delivery by bacteriophage P22. PLoS One 8:13. 10.1371/journal.pone.0070936, PMID: 23951045 PMC3741392

[ref37] Lee C. H. Tsai C. M. (1999). Quantification of bacterial lipopolysaccharides by the purpald assay: measuring formaldehyde generated from 2-keto-3-deoxyoctonate and heptose at the inner core by periodate oxidation. Anal. Biochem. 267, 161–168. 10.1006/abio.1998.2961, PMID: 9918668

[ref38] Leon M. Bastias R. (2015). Virulence reduction in bacteriophage resistant bacteria. Front. Microbiol. 6:343. 10.3389/fmicb.2015.00343, PMID: 25954266 PMC4407575

[ref39] Loeb M. R. (1974). Bacteriophage T4-mediated release of envelope components from *Escherichia-Coli*. J. Virol. 13, 631–641. 10.1128/JVI.13.3.631-641.1974, PMID: 4595901 PMC355348

[ref40] Malge A. Ghai V. Reddy P. J. Baxter D. Kim T. K. Moritz R. L.. (2018). mRNA transcript distribution bias between Borrelia burgdorferi bacteria and their outer membrane vesicles. FEMS Microbiol. Lett. 365:fny135. 10.1093/femsle/fny135, PMID: 29846577 PMC5995203

[ref41] Mangenot S. Hochrein M. Radler J. Letellier L. (2005). Real-time imaging of DNA ejection from single phage particles. Curr. Biol. 15, 430–435. 10.1016/j.cub.2004.12.080, PMID: 15753037

[ref42] Manning A. J. Kuehn M. J. (2011). Contribution of bacterial outer membrane vesicles to innate bacterial defense. BMC Microbiol. 11:258. 10.1186/1471-2180-11-258, PMID: 22133164 PMC3248377

[ref43] Mashburn L. M. Whiteley M. (2005). Membrane vesicles traffic signals and facilitate group activities in a prokaryote. Nature 437, 422–425. 10.1038/nature03925, PMID: 16163359

[ref44] Parent K. N. Erb M. L. Cardone G. Nguyen K. Gilcrease E. B. Porcek N. B.. (2014). OmpA and OmpC are critical host factors for bacteriophage Sf6 entry in Shigella. Mol. Microbiol. 92, 47–60. 10.1111/mmi.12536, PMID: 24673644 PMC4034267

[ref45] Park M. Yoo G. Bong J. H. Jose J. Kang M. J. Pyun J. C. (2015). Isolation and characterization of the outer membrane of *Escherichia coli* with autodisplayed Z-domains. BBA-Biomembranes 1848, 842–847. 10.1016/j.bbamem.2014.12.011, PMID: 25528472

[ref46] Parsegian V. A. Rand R. P. Fuller N. L. Rau D. C. (1986). Osmotic stress for the direct measurement of intermolecular forces. Methods Enzymol. 127, 400–416. 10.1016/0076-6879(86)27032-9, PMID: 3736427

[ref47] Pires D. P. Oliveira H. Melo L. D. R. Sillankorva S. Azeredo J. (2016). Bacteriophage-encoded depolymerases: their diversity and biotechnological applications. Appl. Microbiol. Biotechnol. 100, 2141–2151. 10.1007/s00253-015-7247-0, PMID: 26767986

[ref48] Prevelige P. (2006). “Bacteriophage P22” in The bacteriophages. ed. Calendar R. (Oxford: Oxford University Press), 457.

[ref49] Prokhorov N. S. Riccio C. Zdorovenko E. L. Shneider M. M. Browning C. Knirel Y. A.. (2017). Function of bacteriophage G7C esterase tailspike in host cell adsorption. Mol. Microbiol. 105, 385–398. 10.1111/mmi.13710, PMID: 28513100

[ref50] Reyes-Robles T. Dillard R. S. Cairns L. S. Silva-Valenzuela C. A. Housman M. Ali A.. (2018). Vibrio cholerae outer membrane vesicles inhibit bacteriophage infection. J. Bacteriol. 200:e00792–17. 10.1128/jb.00792-17, PMID: 29661863 PMC6040182

[ref51] Richter W. Vogel V. Howe J. Steiniger F. Brauser A. Koch M. H.. (2011). Morphology, size distribution, and aggregate structure of lipopolysaccharide and lipid a dispersions from enterobacterial origin. Innate Immun. 17, 427–438. 10.1177/1753425910372434, PMID: 20682588

[ref52] Rostol J. T. Marraffini L. (2019). (Ph)ighting phages: how bacteria resist their parasites. Cell Host Microbe 25, 184–194. 10.1016/j.chom.2019.01.009, PMID: 30763533 PMC6383810

[ref53] Rozycki B. Lipowsky R. (2015). Spontaneous curvature of bilayer membranes from molecular simulations: asymmetric lipid densities and asymmetric adsorption. J. Chem. Phys. 142:054101. 10.1063/1.4906149, PMID: 25662630

[ref54] Salmond G. P. C. Fineran P. C. (2015). A century of the phage: past, present and future. Nat. Rev. Microbiol. 13, 777–786. 10.1038/nrmicr03564, PMID: 26548913

[ref55] Schmidt A. Rabsch W. Broeker N. K. Barbirz S. (2016). Bacteriophage tailspike protein based assay to monitor phase variable glucosylations in Salmonella O-antigens. BMC Microbiol. 16:207. 10.1186/s12866-016-0826-0, PMID: 27604475 PMC5015238

[ref56] Schwechheimer C. Kuehn M. J. (2015). Outer-membrane vesicles from gram-negative bacteria: biogenesis and functions. Nat. Rev. Microbiol. 13, 605–619. 10.1038/nrmicro3525, PMID: 26373371 PMC5308417

[ref57] Schwechheimer C. Sullivan C. J. Kuehn M. J. (2013). Envelope control of outer membrane vesicle production in gram-negative bacteria. Biochemistry 52, 3031–3040. 10.1021/bi400164t, PMID: 23521754 PMC3731998

[ref58] Seed K. D. Faruque S. M. Mekalanos J. J. Calderwood S. B. Qadri F. Camilli A. (2012). Phase variable O antigen biosynthetic genes control expression of the major protective antigen and bacteriophage receptor in Vibrio cholerae O1. PLoS Pathog. 8:e1002917. 10.1371/journal.ppat.1002917, PMID: 23028317 PMC3441752

[ref59] Silva J. B. Storms Z. Sauvageau D. (2016). Host receptors for bacteriophage adsorption. FEMS Microbiol. Lett. 363:fnw002. 10.1093/femsle/fnw002, PMID: 26755501

[ref60] Smith D. Tans S. Smith S. Grimes S. Anderson D. Bustamante C. (2001). The bacteriophage phi 29 portal motor can package DNA against a large internal force. Nature 413 748–752. 10.1038/35099581, PMID: 11607035

[ref61] Smith P. K. Krohn R. I. Hermanson G. T. Mallia A. K. Gartner F. H. Provenzano M. D.. (1985). Measurement of protein using bicinchoninic acid. Anal. Biochem. 150, 76–85. 10.1016/0003-2697(85)90442-7, PMID: 3843705

[ref62] Susskind M. M. Botstein D. Wright A. (1974). Superinfection exclusion by P22 prophage in lysogens of *Salmonella-typhimurium*.III. Failure of superinfecting phage DNA to enter SieA+ lysogens. Virology 62, 350–366. 10.1016/0042-6822(74)90398-5, PMID: 4610992

[ref63] Thein M. Sauer G. Paramasivam N. Grin I. Linke D. (2010). Efficient subfractionation of gram-negative bacteria for proteomics studies. J. Proteome Res. 9, 6135–6147. 10.1021/pr1002438, PMID: 20932056

[ref64] Tindall B. J. Grimont P. A. Garrity G. M. Euzéby J. P. (2005). Nomenclature and taxonomy of the genus *Salmonella*. Int. J. Syst. Evol. Microbiol. 55, 521–524. 10.1099/ijs.0.63580-0, PMID: 15653930

[ref65] Toyofuku M. Nomura N. Eberl L. (2019). Types and origins of bacterial membrane vesicles. Nat. Rev. Microbiol. 17, 13–24. 10.1038/s41579-018-0112-2, PMID: 30397270

[ref66] Vidakovic L. Singh P. K. Hartmann R. Nadell C. D. Drescher K. (2018). Dynamic biofilm architecture confers individual and collective mechanisms of viral protection. Nat. Microbiol. 3, 26–31. 10.1038/s41564-017-0050-1, PMID: 29085075 PMC5739289

[ref67] Volgers C. Savelkoul P. H. M. Stassen F. R. M. (2018). Gram-negative bacterial membrane vesicle release in response to the host-environment: different threats, same trick? Crit. Rev. Microbiol. 44, 258–273. 10.1080/1040841x.2017.1353949, PMID: 28741415

[ref68] Wang C. Y. Tu J. G. Liu J. Molineux I. J. (2019). Structural dynamics of bacteriophage P22 infection initiation revealed by cryo-electron tomography. Nat. Microbiol. 4, 1049–1056. 10.1038/s41564-019-0403-z, PMID: 30886360 PMC6533119

[ref69] Whitfield C. Trent M. S. (2014). Biosynthesis and export of bacterial lipopolysaccharides. Annu. Rev. Biochem. 83, 99–128. 10.1146/annurev-biochem-060713-035600, PMID: 24580642

[ref70] Wu E. L. Fleming P. J. Yeom M. S. Widmalm G. Klauda J. B. Fleming K. G.. (2014). *E. coli* outer membrane and interactions with OmpLA. Biophys. J. 106, 2493–2502. 10.1016/j.bpj.2014.04.024, PMID: 24896129 PMC4052237

