# Supplementary material for: In vitro Analysis of O-Antigen-Specific Bacteriophage P22 Inactivation by Salmonella Outer Membrane Vesicles
Source: Front Microbiol. 2020 Sep 24;11:510638. doi: 10.3389/fmicb.2020.510638 (PMC7541932; doi:10.3389/fmicb.2020.510638)
Supplement: Supplementary file 1 [file Data_Sheet_1.pdf]

Supplementary Information for

In vitro analysis of O-antigen specific bacteriophage P22 inactivation by *Salmonella* outer membrane vesicles

Mareike S. Stephan[1], Nina K. Broeker[1], Athanasios Saragliadis[2], Norbert Roos[2], Dirk Linke[2], Stefanie Barbirz[1*]

[1] Physical Biochemistry, Department for Biochemistry and Biology, University of Potsdam, 14476 Potsdam, Germany

[2] Department of Biosciences, University of Oslo, 0371 Oslo, Norway

*** Correspondence:**
Corresponding Author
barbirz@uni-potsdam.de

Table of contents

Figure S1: Dynamic light scattering analysis of OMV size distribution 2

Figure S2: TEM analysis of OMV preparations without pre-lysis treatment with DNAse I and lysozyme. 3

Figure S3: Characterization of OMVs from membrane accumulation 4

Figure S4: SDS-PAGE analysis of OMVs with glycan staining of LPS fraction 5

Figure S5: Protein content of different outer membrane vesicle preparations analyzed by SDS-PAGE 6

Figure S6: Exemplary EM image for semi-quantitative estimates of frequent appearances of OMV-bound phage 7

Figure S7: DNA ejection from bacteriophage P22 with OMVs from different sources and preparation methods 8

Figure S8: DNA ejection from bacteriophage P22 at different OMV concentrations and with the addition of LPS 9

Figure S9: DNA ejection from bacteriophage P22 triggered by OMV or LPS 10

Figure S10: DNAse accessibility of DNA released after P22 was triggered with either OMV or LPS at different osmotic pressures 11

Figure S1: Dynamic light scattering analysis of OMV size distribution

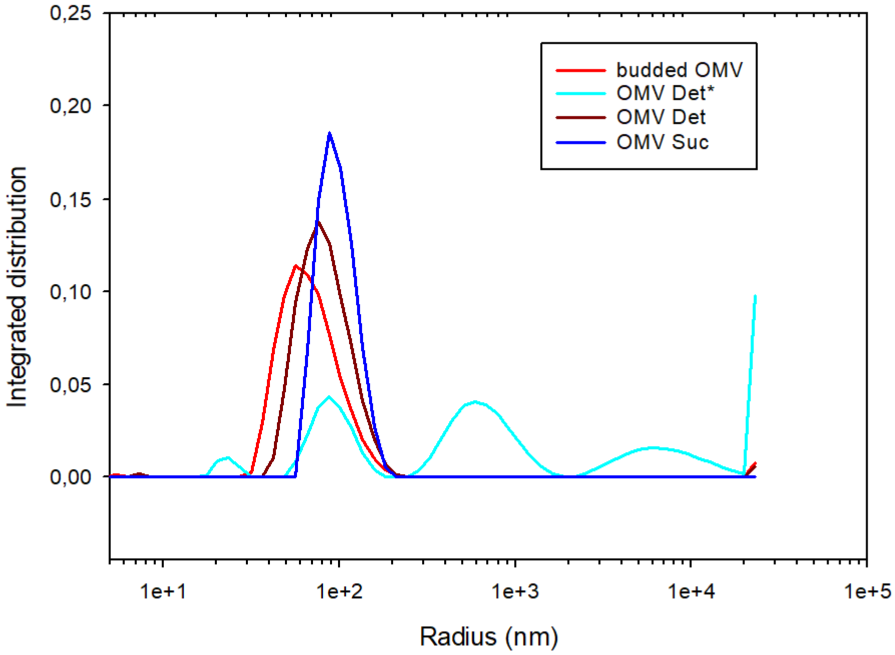

Dynamic light scattering (DLS) measurements were performed at 23 °C at a scattering angle of 90°, utilizing a custom-built instrument, equipped with a continuous wave laser (Cobolt 532 nm, 0.5 W) and a high quantum yield avalanche photodiode (Physikalische Biochemie, University of Potsdam). A primary data accumulation interval T_{acc} of 10 s was applied (Gast *et al.* (1997), *Eur. Biophys. J.* 25, 211-219. doi: 10.1007/s002490050033). The translational diffusion coefficients D were calculated from the measured autocorrelation functions using CONTIN (Provencher, S.W. (1982), *Comp. Phys. Commun.* 27(3)**,** 229-242. doi: 10.1016/0010-4655(82)90174-6). The diffusion coefficients were transformed into Stokes radii R_S by application of the Stokes-Einstein equation (1). Water and ethylene glycol viscosities were determined with an Ubbelohde-type viscometer (Viscoboy-2, Lauda, Germany). Stokes radii are presented in mean-rated radius distributions. For a legend of the different sample preparation methods refer to Table 1 in the main manuscript.

$$R_s = \frac{k_B \cdot T}{6 \cdot \pi \cdot \eta \cdot D} \qquad (1)$$

k_B *Boltzmann constant*
R_s *Stokes radius*
T *Temperature in Kelvin*
η *Solvent viscosity*
D *Diffusion coefficient*

Figure S2: TEM analysis of OMV preparations without pre-lysis treatment with DNAse I and lysozyme.

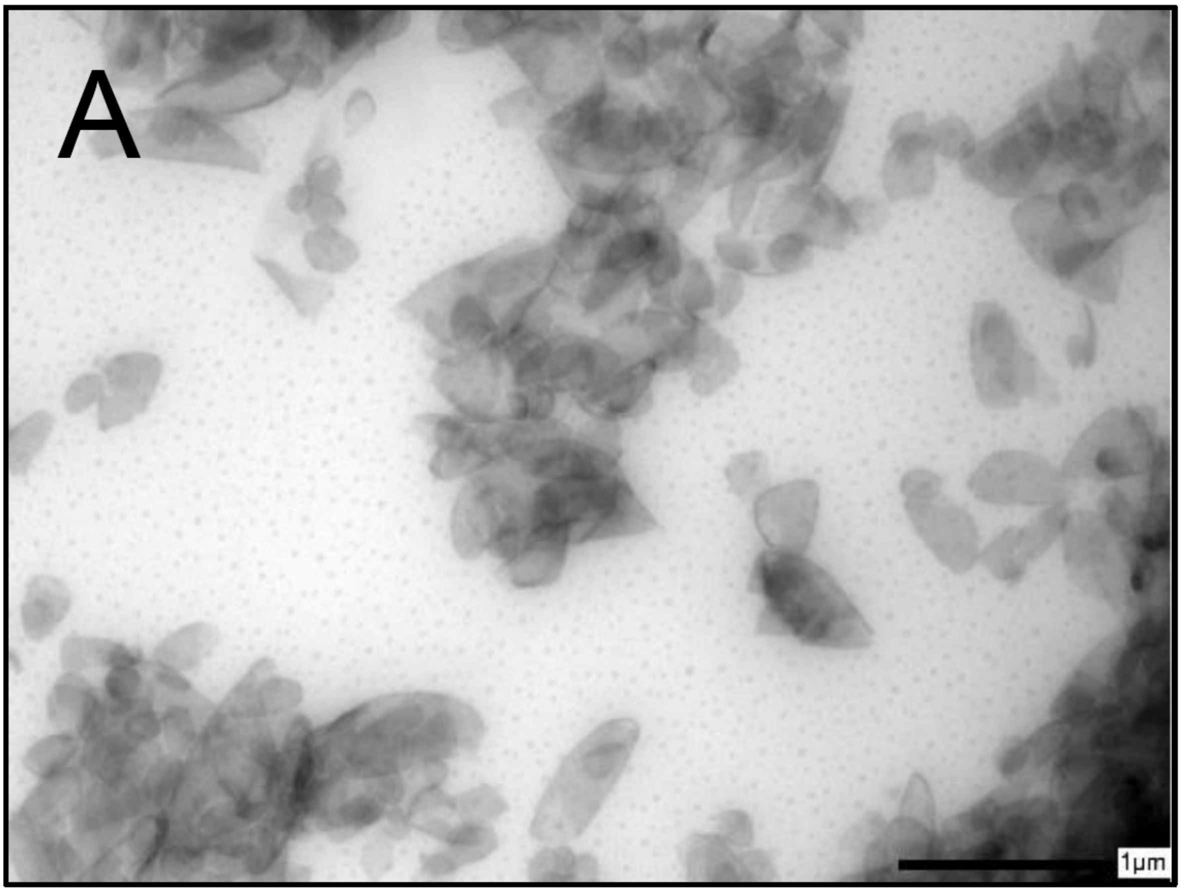

OMV (A) were embedded in methylcellulose and stained using uranyl acetate. The vesicles showed radii of 50 to 425 nm.

Figure S3: Characterization of OMVs from membrane accumulation

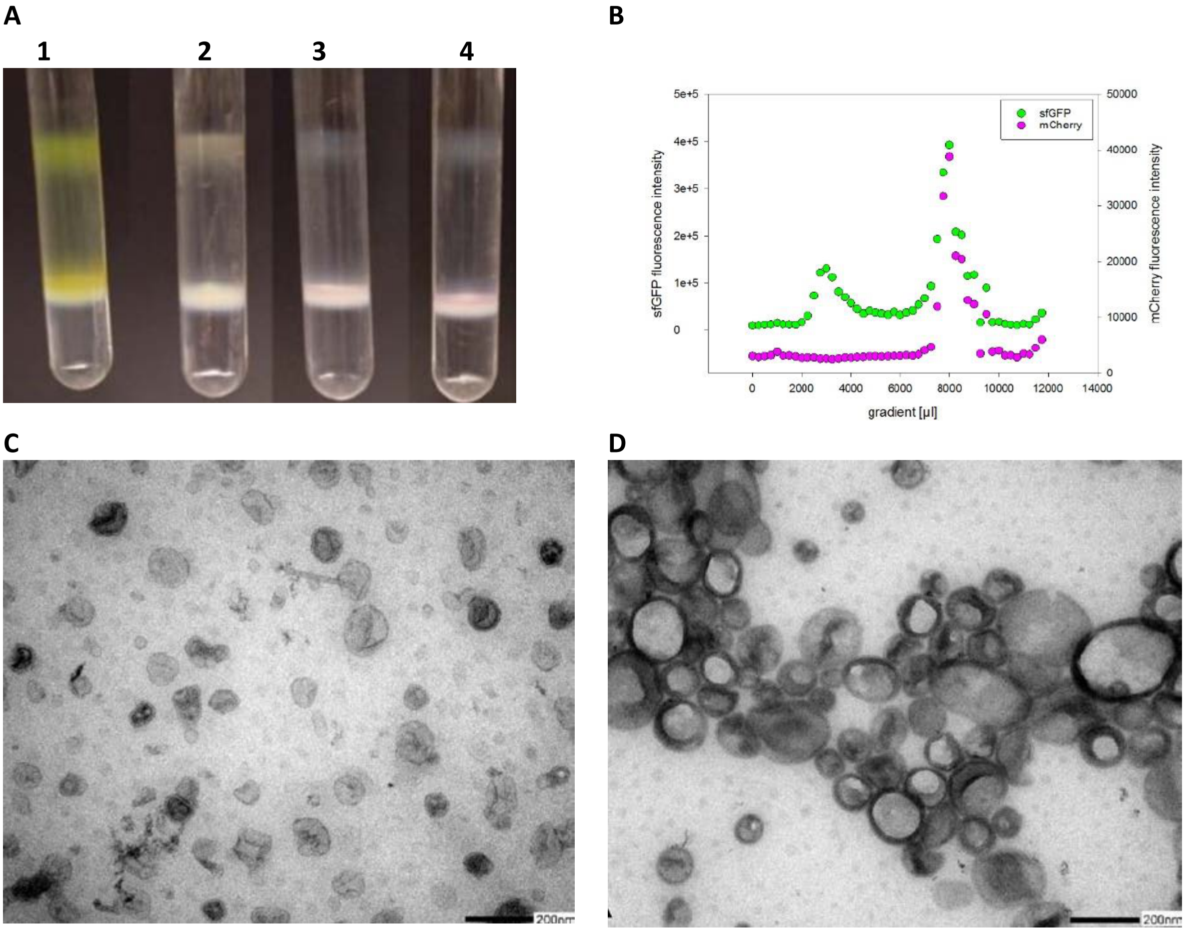

Salmonella Typhimurium MvP103 were transformed with plasmids containing either OmpA-mCherry (outer membrane marker) or the pf3-sfGFP (inner membrane marker) (Winther, 2015).

A. Sucrose density gradients of membrane preparations of cell. (1) pASK IBA2 pf3-sfGFP. The pf3-sfGFP localizes in the inner membrane (upper band) but is also present in the outer membrane fraction (lower band). (2) Empty plasmid (pASK IBA 2). (3) pASK IBA2 OmpA-mCherry pf3-sfGFP. The pf3-sfGFP localizes in the inner membrane (upper band) but is also present in the outer membrane fraction (lower band). (4) pASK IBA2 OmpA-mCherry The OmpA-mCherry localizes in the outer membrane (lower band).

B. sfGFP and mCherry fluorescence intensities in the sucrose density gradient after separation of inner and outer membrane.

C, D: TEM images of inner membrane fraction (C) and outer membrane fraction (D) after sucrose gradient separation. Bars represent 200 nm.

Winther, A.R. Fluorescent Biomarkers for Membrane Separation. Master Thesis University of Oslo, Oslo, 2015. http://urn.nb.no/URN:NBN:no-53460

Figure S4: SDS-PAGE analysis of OMVs with glycan staining of LPS fraction

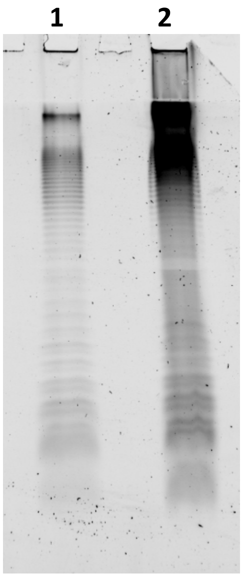

SDS-PAGE analysis of OMVs from *Salmonella* Typhimurium AroA::Tn10 iroBC::kan obtained by membrane accumulation and sucrose gradient purification. LPS in the gel was visualized with the Pro-Q® Emerald 300 glycan-specific stain (Thermo Fisher Scientific, Dreieich, Germany) according to the manufacturer's instructions. 3 µg (lane 1) or 30 µg (lane 2) LPS were applied, respectively.

Figure S5: Protein content of different outer membrane vesicle preparations analyzed by SDS-PAGE

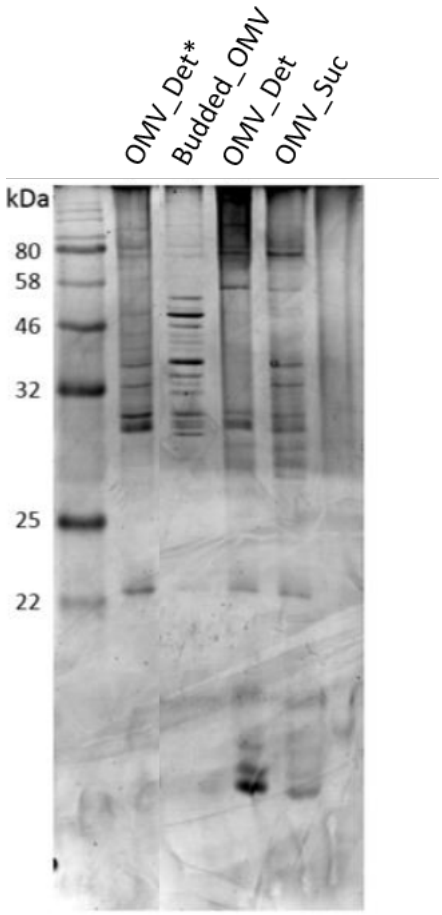

12.5 % Polyacrylamide gel loaded with different OMV preparations (10 µg per lane) and silver stained. 10 µg purified LPS were loaded as control (far right lane).

Figure S6: Exemplary EM image for semi-quantitative estimates of frequent appearances of OMV-bound phage

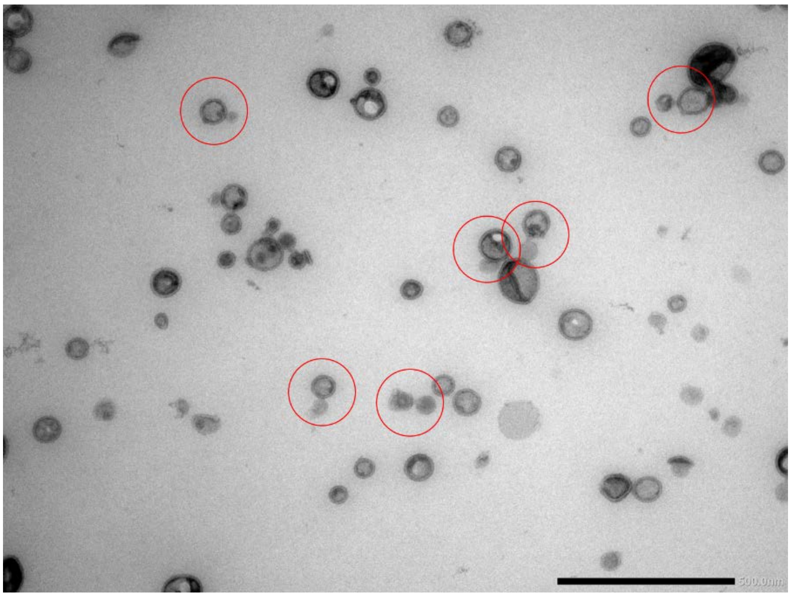

Typical overview of negative stain EM analysis of phage P22 in presence of OMVs (here prepared with the sucrose gradient method).

Figure S7: DNA ejection from bacteriophage P22 with OMVs from different sources and preparation methods

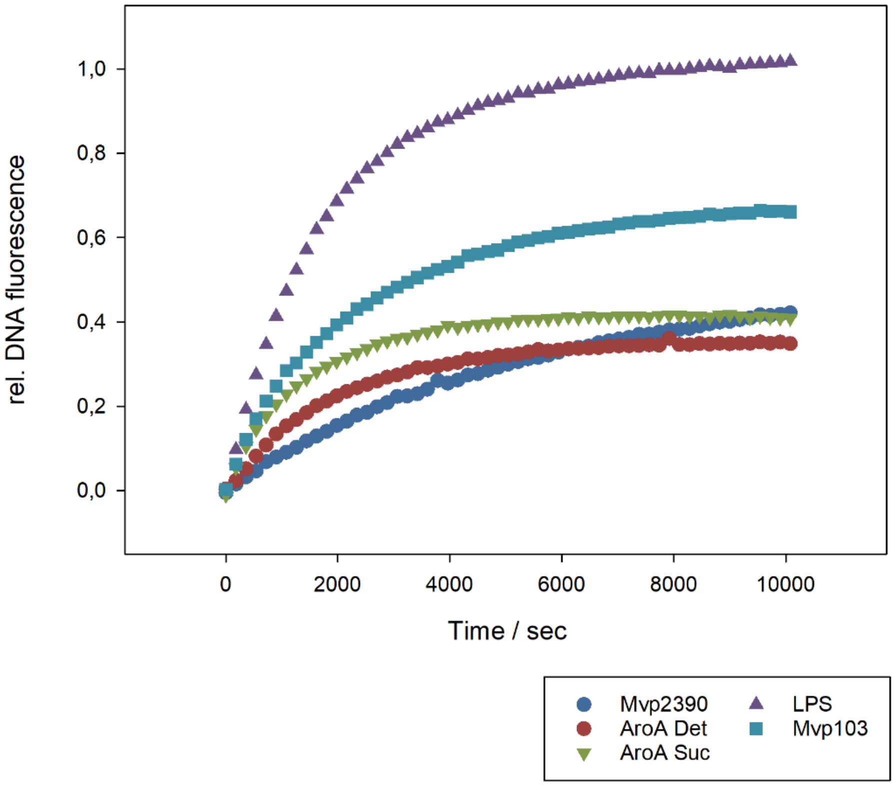

Incubation of $4*10^9$ pfu/ml P22 phages with OMVs at 37 °C in presence of a fluorescent DNA-binding dye (1 µM Yo-Pro) and with 10 µg ml^{-1} LPS (violet triangles up) or OMVs naturally budded from *Salmonella* Typhimurium Mvp103 (blue squares) or Mvp2390 (blue circles), or OMVs obtained from *S.* Typhimurium AroA with membrane accumulation and sucrose gradient purification (green triangles down) or obtained by detergent separation of inner membranes (red circles). All OMVs were present as 10 µg ml^{-1} LPS equivalents.

Figure S8: DNA ejection from bacteriophage P22 at different OMV concentrations and with the addition of LPS

A

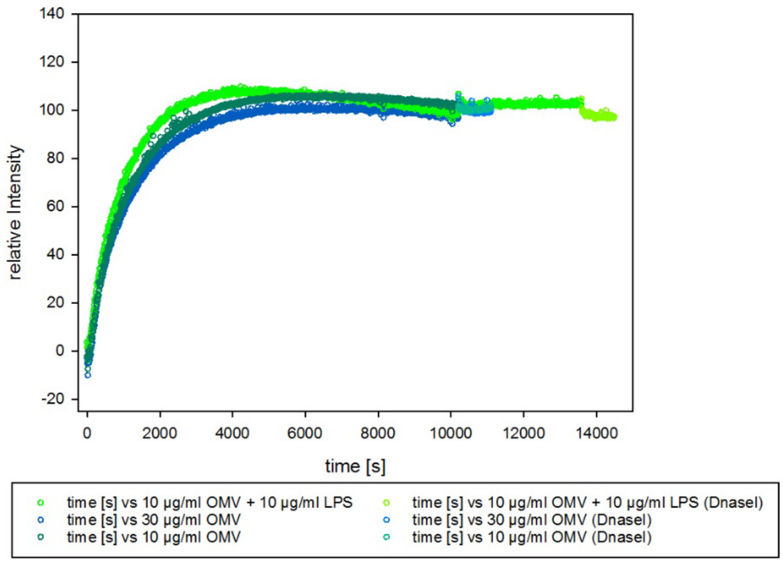

B

Incubation of $4*10^9$ pfu/ml P22 phages with OMVs at 37 °C in presence of a fluorescent DNA-binding dye (1 µM Yo-Pro). (A) 10 µg ml^{-1} (dark green) or 30 µg ml^{-1} (blue) OMVs. After 170 min, DNase I (10 µg ml^{-1}) was added. In one sample, after 170 min purified LPS was added (10 µg ml^{-1}) to probe whether ejection competent phages had remained in solution after signal equilibration.

Figure S9: DNA ejection from bacteriophage P22 triggered by OMV or LPS

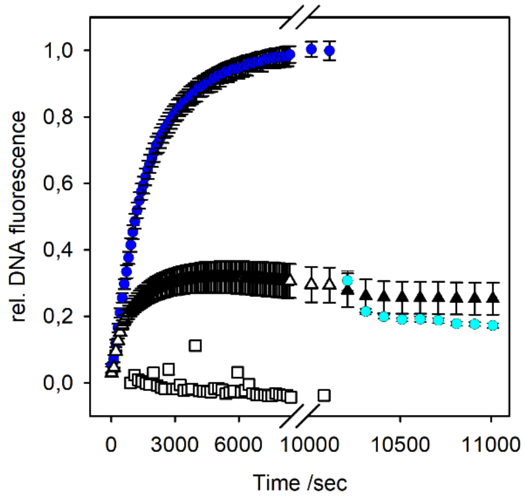

Incubation of $4*10^9$ pfu/ml P22 phages at 37 °C in presence of a fluorescent DNA-binding dye (1 µM Yo-Pro) with 10 µg ml^{-1} LPS (blue circles) or OMV (white triangles; 10 µg ml^{-1} LPS equivalents, see Materials and Methods). After 170 min DNaseI (10 µg ml^{-1}) was added to the ejected phages, either LPS-triggered (cyan circles) or OMV-triggered (black triangles). Plot shows mean values of three technical replicates with error bars representing the standard deviation. As a control, 10 µg ml^{-1} OMV LPS equivalents were incubated overnight with 10 µg ml^{-1} P22TSP at 37 °C prior to the ejection experiment (white squares).

Figure S10: DNAse accessibility of DNA released after P22 was triggered with either OMV or LPS at different osmotic pressures

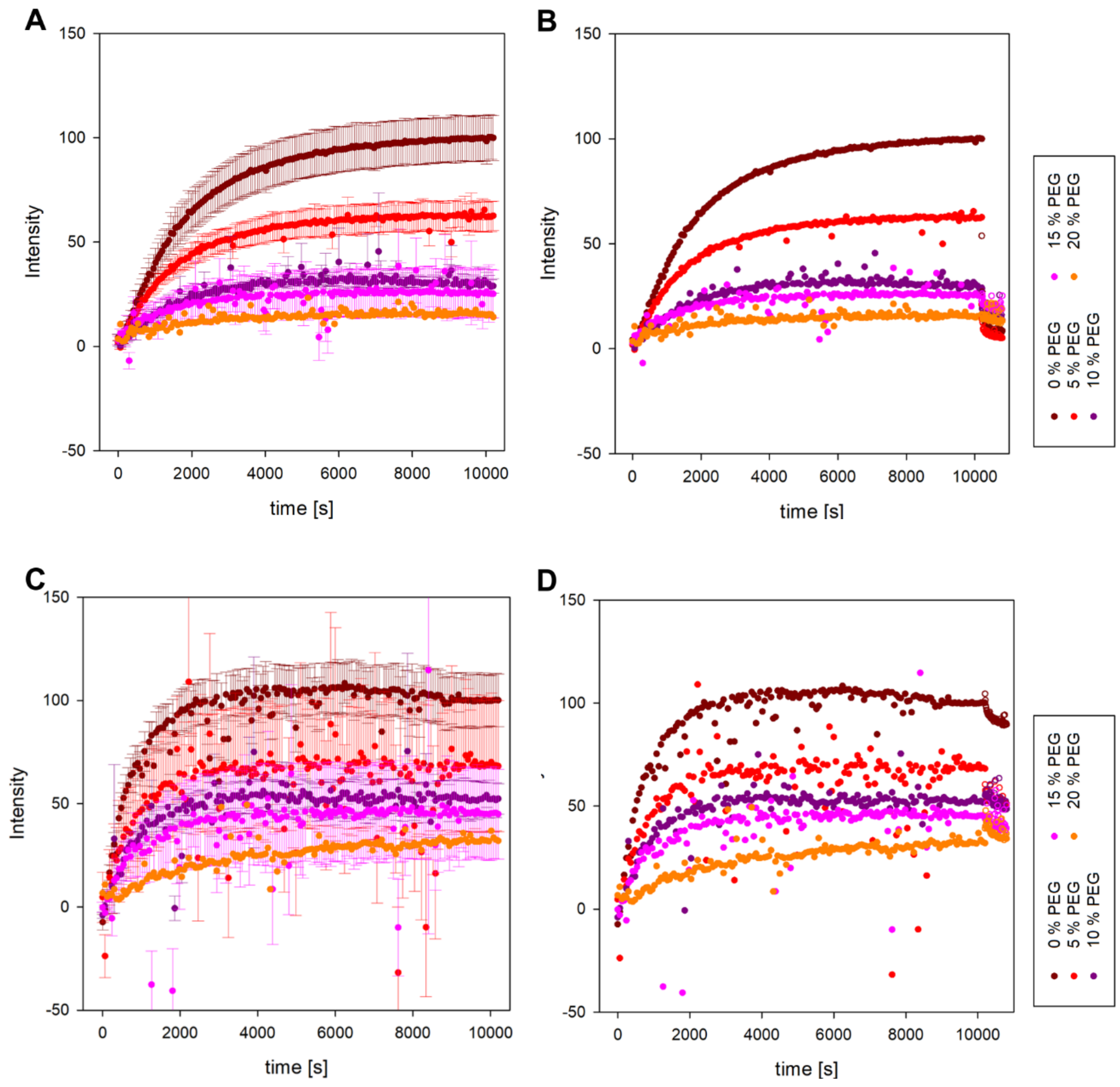

DNA release from bacteriophage P22 triggered by OMV or LPS at different solution osmotic pressures. Incubation of $4*10^9$ pfu/ml P22 phages at 37 °C in presence of fluorescent DNA-binding dye Yo-Pro (1 µM) with (A, B) 10 µg ml^{-1} LPS or (C,D) OMV (10 µg ml^{-1} LPS equivalents) at increasing PEG 8000 concentrations. Plots show mean values of three technical replicates with standard deviations (bars in A, C). The values for 20 % PEG have been determined in a single measurement only and are shown for comparison. After 10000 sec, DNAse was added to the solutions (B, D; error bars were omitted for clarity).
